# Die Bedeutung der Strahlentherapie im Kontext immer effektiver werdender Systemtherapien beim metastasierten NSCLC – Hinweise aus einer Rezidivanalyse nach Immuncheckpointinhibitoren

**DOI:** 10.1007/s00066-021-01864-4

**Published:** 2021-10-26

**Authors:** Philipp Schubert, Rainer Fietkau, Markus Hecht

**Affiliations:** Univ.-Strahlenklinik, Universitätsstraße 27, 91054 Erlangen, Deutschland

## Hintergrund und Ziele

Die Applikation von Immuncheckpoint-Inhibitoren bei lokal fortgeschrittenen und metastasierten NSCLC verlängert das Gesamtüberleben (OS) sowie das progressionsfreie Überleben (PFS) erheblich. Ein dauerhaftes Ansprechen auf die Therapie mit bis zu 5 Jahren ist durchaus möglich. Allerdings kann es bei diesen Langzeitüberlebenden im Verlauf auch wieder zur Progression kommen, da die Tumoren dieser Patienten vermutlich eine Therapieresistenz gegenüber der Immuntherapie entwickeln („acquired resistance“). Dieser Mechanismus ist bereits beim NSCLC nach Tyrosinkinaseinhibitortherapie bekannt und kann möglicherweise auf Patienten unter Immuntherapie übertragen werden. Daher untersuchten Heo et al. in einer retrospektiven Analyse das Progressmuster nach anhaltender Therapieantwort mit dem Ziel, Therapiestrategien nach Progress bei diesen Patienten zu definieren.

## Patienten und Methoden

In dieser retrospektiven Analyse wurden Patienten mit NSCLC unter Anti-PD-1- oder PD-L1-Therapie ausgewertet, insbesondere mit radiologischen Untersuchungen bei Patienten, die länger als 6 Monate auf eine Immuntherapie ansprachen. Zwei unabhängige Radiologen beurteilten diese Untersuchungsergebnisse im Verlauf nach den RECIST‑1.1‑Kriterien. Zur Einordnung des Progressionsmusters wurden Subgruppen mit intrathorakaler, extrathorakaler und hepatischer Progression gebildet. Das PFS wurde vom Zeitpunkt der Immuntherapieinitiierung bis zum Progress nach RECIST 1.1, Tod oder letzten Kontakt definiert.

## Ergebnisse

125 Patienten mit anhaltender Therapieantwort von mehr als 6 Monaten wurden gescreent. Hiervon zeigten sich die Karzinome von 63 Patienten progredient. Der Progress wurde als erworbene Resistenz klassifiziert. Die Resistenz entwickelte sich im Mittel nach 10,7 Monaten. Die Mehrzahl der Patienten (52) waren lediglich oligoprogredient, d. h. mit 1 (73 %) oder 2 (9,5 %) progredienten Läsionen. Mehr als 3 Lokalisationen waren lediglich bei 11 Patienten (17,5 %) festzustellen. Hauptsächlich waren diese Tumorprogressionen in der Lunge zu finden (60,3 %), gefolgt von Lymphknotenmetastasen (49,2 %). Hiervon bildeten 20,6 % neue Läsionen aus; in der Mehrzahl waren mit 68,3 % bereits bestehende Läsionen progredient. Oligoprogrediente Patienten zeigten eine hohe Anzahl (27 %) von Progressen isoliert als Lymphknotenmetastasen bei im Übrigen weiteren, stabilen Läsionen. Das mediane OS nach erworbener Resistenz betrug 10,9 Monate. Bei oligoprogredienten Patienten war das mediane Gesamtüberleben mit 18,9 Monaten (95 %-CI 10,6–NR) signifikant besser als bei multiprogredienten mit 8,8 Monaten (95 %-CI 5,7–NR; *p* = 0,04). Die Überlebensrate war zwischen Patienten mit einer Fortführung der Immuntherapie und einer Einleitung einer Chemotherapie nicht unterschiedlich (*p* = 0,723). Ebenso ergab sich im medianen OS kein Vorteil für eine Lokaltherapie im Vergleich zur Einleitung einer Chemotherapie oder Fortführung der Immuntherapie (27,4 vs. 23,2 vs. 29,1 Monate; *p* = 0,787).

## Schlussfolgerung der Autoren

Nach erworbener Resistenz gegen die Immuntherapie zeigten die meisten Patienten eine Progression im Sinne einer Oligoprogression mit vermehrter Metastasierung in Lymphknoten. Eine Lokaltherapie dieser Lokalisationen mit anschließender Fortführung der Immuntherapie könnte in solchen Fällen eine wirksame Therapieoption sein.

## Kommentar

Der Einsatz von Immuncheckpointinhibitoren beim NSCLC ist mittlerweile aus dem klinischen Alltag nicht mehr wegzudenken. Trotz unbestrittener Effektivität hat auch diese Therapie ihre Grenzen. Die vorliegende Analyse greift dieses Problem auf.

Die Frage, wie eine Oligoprogression am besten behandelt wird bzw. sich überhaupt verhindern lässt, war bereits Gegenstand verschiedener, auch prospektiver Erhebungen. Erste Phase-II-Studien weisen darauf hin, dass bei ähnlichem Nebenwirkungsprofil eine Kombination von System- und Strahlentherapie zu Vorteilen im progressionsfreien und Gesamtüberleben führen kann, verglichen mit einer alleinigen Systemtherapie. Dies trifft nicht nur auf die Kombination mit Chemo-, sondern auch mit der Immuntherapie zu [1, 2].

Das Progressionsmuster in der vorliegenden Analyse zeigte gewisse Charakteristika: Zum einen fiel auf, dass die Mehrzahl der Patienten nach einer anhaltenden Therapieantwort von > 6 Monaten an nur wenigen Stellen, konkret an bis zu zwei, progredient war, und zwar in der Mehrzahl an bereits bekannten Lokalisationen. Das hier mehrheitlich festzustellende Oligoprogressionsmuster bietet somit die ideale Grundlage für eine konsequente Lokaltherapie.

Des Weiteren ist festgehalten, dass das Überleben der oligoprogredienten Gruppe erwartungsgemäß signifikant länger war im Vergleich mit der systemisch progredienten Gruppe. Der postulierte Mechanismus dahinter könnte sein, dass eine Tumorzelle unter „Kontrolle“ des Immunsystems erst dann in der Lage ist, andere Organsysteme zu befallen, sobald diese bspw. durch Hochregulation von alternativen Immuncheckpoints wie CTLA‑4 oder TIM‑3 einen neuen Immunphänotyp ausbildet, der das Wirtsimmunsystem umgehen kann [3]. Diese Patienten würden sich gut für die Einleitung einer lokalen Therapie der progredienten Läsionen mit Strahlentherapie eignen, um die neuen Phänotypen zu kontrollieren. In neueren klinischen Analysen zeigten sich signifikante Vorteile bei Bestrahlung aller Läsionen gegenüber der Bestrahlung nur eines Teils der progredienten Läsionen [4]. Wenngleich in der vorliegenden Analyse die lokal bestrahlten Patienten mit Progress kein signifikant längeres Überleben zeigten, muss dieses sicherlich vor dem Hintergrund der niedrigen Patientenzahl in dieser Gruppe (*n* = 11) kritisch betrachtet werden, welche es nicht erlaubt, eine entsprechende statistisch sinnvolle Differenzierung der Subgruppen durchzuführen.

Dass die Strahlentherapie auch immunmodulatorische Effekte hat, ist lange bekannt. Veränderungen des Tumormikromilieus, insbesondere des immunogenen Zelltods, und das Freisetzen von Signalmolekülen zur indirekten Aktivierung des Immunsystems, ebenso die direkte Aktivierung von Immunzellen, sind hierbei von Interesse [5]. Im Rahmen dessen ist der isolierte Progress an Lymphknoten zu interpretieren. Hier scheint ein verändertes Tumormikromilieu innerhalb des befallenen Lymphknotens die oben genannten Immun-Escape-Strategien zu begünstigen und zur Entstehung der erworbenen Resistenz auf die Immuntherapie beizutragen.

## Fazit

Das Progressionsmuster bei metastasierten NSCLC-Patienten, die länger als 6 Monate mit PD-1/PD-L1-Inhibitoren behandelt werden, zeigt hauptsächlich ein Fortschreiten der Erkrankung in bereits vorhandenen Läsionen, und dies mehrheitlich im Sinne einer Oligoprogression. Eine konsequente Lokaltherapie kann selbst in metastasierten Stadien ein wichtiger Pfeiler bei immer effektiver werdenden Systemtherapien bestimmter Tumorerkrankungen sein.

Aus Sicht der Radioonkologie bleiben aber weitere Fragen offen, z. B.: Wann ist der optimale Zeitpunkt für die Bestrahlung, bereits zu Beginn der Immuntherapie oder erst im weiteren Verlauf, also im Falle einer Oligoprogression? Auch die Zahl der zu behandelnden Lokalisationen, die Fraktionierung der Therapie und die Einzeldosis müssen in weiteren Studien noch diskutiert werden.

*Philipp Schubert, Rainer Fietkau, Markus Hecht, Erlangen*
